# Unexpected running perturbations: Reliability and validity of a treadmill running protocol with analysis of provoked reflex activity in the lower extremities

**DOI:** 10.3389/fspor.2023.1129058

**Published:** 2023-03-15

**Authors:** Andrew Quarmby, Mina Khajooei, Philip Kurtz, Jakob Henschke, MyoungHwee Kim, Frank Mayer, Tilman Engel

**Affiliations:** University Outpatient Clinic, Sports Medicine & Sports Orthopaedics, University of Potsdam, Potsdam, Germany

**Keywords:** running, perturbation, EMG, reliability, stumbling, reflexes, split-belt treadmill, gait

## Abstract

**Introduction:**

Balance is vital for human health and experiments have been conducted to measure the mechanisms of postural control, for example studying reflex responses to simulated perturbations. Such studies are frequent in walking but less common in running, and an understanding of reflex responses to trip-like disturbances could enhance our understanding of human gait and improve approaches to training and rehabilitation. Therefore, the primary aim of this study was to investigate the technical validity and reliability of a treadmill running protocol with perturbations. A further exploratory aim was to evaluate the associated neuromuscular reflex responses to the perturbations, in the lower limbs.

**Methods:**

Twelve healthy participants completed a running protocol (9 km/h) test-retest (2 weeks apart), whereby 30 unilateral perturbations were executed via the treadmill belts (presets:2.0 m/s amplitude;150 ms delay (post-heel contact);100ms duration). Validity of the perturbations was assessed via mean ± SD comparison, percentage error calculation between the preset and recorded perturbation characteristics (PE%), and coefficient of variation (CV%). Test-retest reliability (TRV%) and Bland-Altman analysis (BLA; bias ± 1.96 * SD) was calculated for reliability. To measure reflex activity, electromyography (EMG) was applied in both legs. EMG amplitudes (root mean square normalized to unperturbed strides) and latencies [ms] were analysed descriptively.

**Results:**

Left-side perturbation amplitude was 1.9 ± 0.1 m/s, delay 105 ± 2 ms, and duration 78 ± 1 ms. Right-side perturbation amplitude was 1.9 ± 0.1 m/s, delay 118 ± 2 ms, duration 78 ± 1 ms. PE% ranged from 5–30% for the recorded perturbations. CV% of the perturbations ranged from 19.5–76.8%. TRV% for the perturbations was 6.4–16.6%. BLA for the left was amplitude: 0.0 ± 0.3m/s, delay: 0 ± 17 ms, duration: 2 ± 13 ms, and for the right was amplitude: 0.1 ± 0.7, delay: 4 ± 40 ms, duration: 1 ± 35 ms. EMG amplitudes ranged from 175 ± 141%–454 ± 359% in both limbs. Latencies were 109 ± 12–116 ± 23 ms in the tibialis anterior, and 128 ± 49-157 ± 20 ms in the biceps femoris.

**Discussion:**

Generally, this study indicated sufficient validity and reliability of the current setup considering the technical challenges and limitations, although the reliability of the right-sided perturbations could be questioned. The protocol provoked reflex responses in the lower extremities, especially in the leading leg. Acute neuromusculoskeletal adjustments to the perturbations could be studied and compared in clinical and healthy running populations, and the protocol could be utilised to monitor chronic adaptations to interventions over time.

## 1. Introduction

Maintenance of balance during human locomotion is crucial to avoid falls and potential injuries (1–6). This has generated substantial interest in the biomechanical study of postural motor control strategies (2, 7–9), usually in an experimental setup whereby a motor task, is intentionally perturbed and the physiological responses are simultaneously measured (2, 6, 8–11). External perturbations can be experimentally induced with a variety of techniques, and these were recently reviewed (8). Tokur et al., (2020) (8) identified a range of studies which artificially simulated external perturbations during walking, including uneven ground changes (12), external forces (13), and swing phase obstacles (14). The authors of this study (8) suggest that running is a less investigated motor task in this domain (8), despite the increasing popularity of the sport and associated high risk of running-related injuries (15). Some studies have experimented with uneven ground (16) and alterations in running surface (17) during running tasks. A study by Ellis et al., (2014) (18) was able to report on levels of muscle contribution during running when varying levels of constant load were applied *via* a horizontal pulley apparatus, however, this approach did not allow for a provocation of reflex activity. A separate study by Haudum et al., (2012) (19) simulated perturbations utilising tubes attached to the ankles and lower back during running at a velocity of 2.9 m/s, and reported observable changes in muscle activity and long-term adaptations. However, it is likely that participants were able to predict the onset of these external perturbations, which almost certainly would have affected the physiological responses. None of the previously identified studies were able to produce unexpected, rapid, and powerful perturbations to provoke reflex responses, as already accomplished in walking protocols (10, 20, 21). An understanding of reflexes is particularly pertinent regarding fall risk (7, 22) and might improve the management of clinical conditions, such as in musculoskeletal disorders (23), cognitive impairments (24), and neurological conditions (25).

Instrumented treadmills offer the opportunity to deliver short-timed stimuli perturbations, which are both powerful and unexpected (10, 20, 21, 26), and such characteristics are essential to elicit valid reflex responses (20–22). However, the validity and reliability of such protocols are rarely reported (10, 20, 21), and the orchestration of such systems is technically challenging. Recently, a running protocol was developed to initiate distal decelerative perturbations to the right leg *via* instrumented treadmill belts, during the mid-stance of gait (26). The protocol was designed to simulate a situation in which a runner might trip whilst running overground, and the perturbations were able to elicit reflex responses with appropriate validity. Whilst the ecological validity of the protocol could be questioned, the experimental design allows for the controlled execution of perturbations within a laboratory setting, which could yield interesting insights into human biomechanics during running (27). However, the reliability of the experimental setup remains untested, as does the validity of the perturbations executed by indirect triggering.

Additionally, the mechanism by which humans maintain an upright posture when gait is perturbed is still not fully understood (8). For instance, the leg directly perturbed is thought to play an active role in compensation (19, 28), but the role of the swing trailing leg has received less research attention (27). A recent study investigated kinematic compensations in response to the identical perturbed running protocol as the one utilised in the present study (27), and it appears that the swing trailing leg was most involved in the active kinematic compensation and maintenance of postural control. However, muscle activity was not reported within this trial, which makes it difficult to fully interpret the findings. A comprehensive understanding of motor control during human gait has many potential applications, and enhanced knowledge of the fundamental neurophysiology might be relevant for a wide-range of fields (29, 30). For example, return to running rehabilitation and sports performance (31, 32), contributions to algorithmic programming in wearable technologies that seek to measure human gait accurately and reliably (33), and even implementation in robotic-assisted gait rehabilitation (34). Without the pre-requisite basic scientific knowledge of patterns and variations in human motor control, attempts to develop satisfactory solutions are likely to be inadequate. Consequently, an investigation into the electromyographic (EMG) profiles of the lower-limb musculature in response to perturbed running could reveal useful information on the motor control strategies of humans. EMG is capable of characterising human performance *via* the capture of muscle activity during different movement tasks (35), and enables unique understanding of motor control during human gait, for example in the study of inter-limb and inter-muscle coordination (11, 23, 36), muscle synergies (30), and neuromuscular reflex responses (23, 30, 37, 38). Investigation of reflex responses may be particularly relevant in the comprehension of fall risk and associated injury (2, 4), and increased understanding could help in the mitigation of this risk by informing training and rehabilitation. Whilst previous studies have investigated reflex responses during walking and running gait (10, 19, 20, 30), seemingly no previous studies have attempted to deliver rapid and powerful distal belt perturbations during running, that are both valid and reliable, and able to evoke reflexes in the lower-limbs. The accuracy and repeatability of such protocols is critical so that robust interpretations of the data can be ascertained, especially if applying such measurements in longitudinal repeat-measures study designs (39). Despite this, reliability analysis is rarely conducted within studies related to perturbed gait (8, 21) and research is required to examine whether such tasks and the corresponding reactions are reproducible.

Therefore, the primary aim of this study was to investigate the technical reliability of a treadmill running protocol with perturbations, specifically designed to provoke neuromuscular reflexes. The study was performed in a test-retest design. A secondary aim was to test the validity of the indirectly triggered perturbations. A third exploratory aim was to analyse the corresponding neuromuscular reflex activity resulting from the perturbations. It should be stressed that this third aim was merely tested to inform whether the protocol elicited reflex responses or not, and if the protocol can therefore be deemed as valid. The further exploratory analysis of inter-limb differences was performed to inform future research directions and cannot be taken as robust scientific evidence. It was hypothesised that the protocol would be reliable according to a quantified statistical approach, that the validity of the indirectly triggered perturbations would be similar to previously published data on the directly triggered perturbations (26), and that the protocol would provoke a quantifiable increase in neuromuscular reflex activity compared to unperturbed running, as measured by EMG. Additionally, an exploratory analysis of the EMG data was conducted, to investigate the amplitude and variability of reflex muscle activity both between independent trials on a test-retest basis and between legs within-subject. As a final exploratory analysis, onset latencies of muscle activity in response to the perturbations were calculated and the reliability of this assessment was tested between the two measurement timepoints. This exploratory analysis should be treated as such, and was not supposed to provide robust scientific findings, but simply suggest an avenue for interesting future research directions.

## 2. Materials and methods

### 2.1. Participants

A convenience sample of twelve participants were tested in the current study (Males = 7, Females = 5; 32 ± 6 years; 72 ± 13 kg; 176 ± 10 cm), in a test-retest design. Participants were recruited based upon the following information: inclusion criteria—(1) 18–50 years of age, (2) Experience in running on a treadmill; exclusion criteria—(1) Any musculoskeletal, vascular, or neurological injury, surgery or illness within the last six months, (2) Acute infection/cold, (3) Severe and debilitating pain with physical activity, (4) Pregnancy. All participants underwent a medical examination, conducted by a physician. Participants were informed about the scope of the study, before giving written informed consent. The study was also approved by the local University of Potsdam ethics committee (application number: 25/2021).

### 2.2. Technical setup

**Figure 1:**
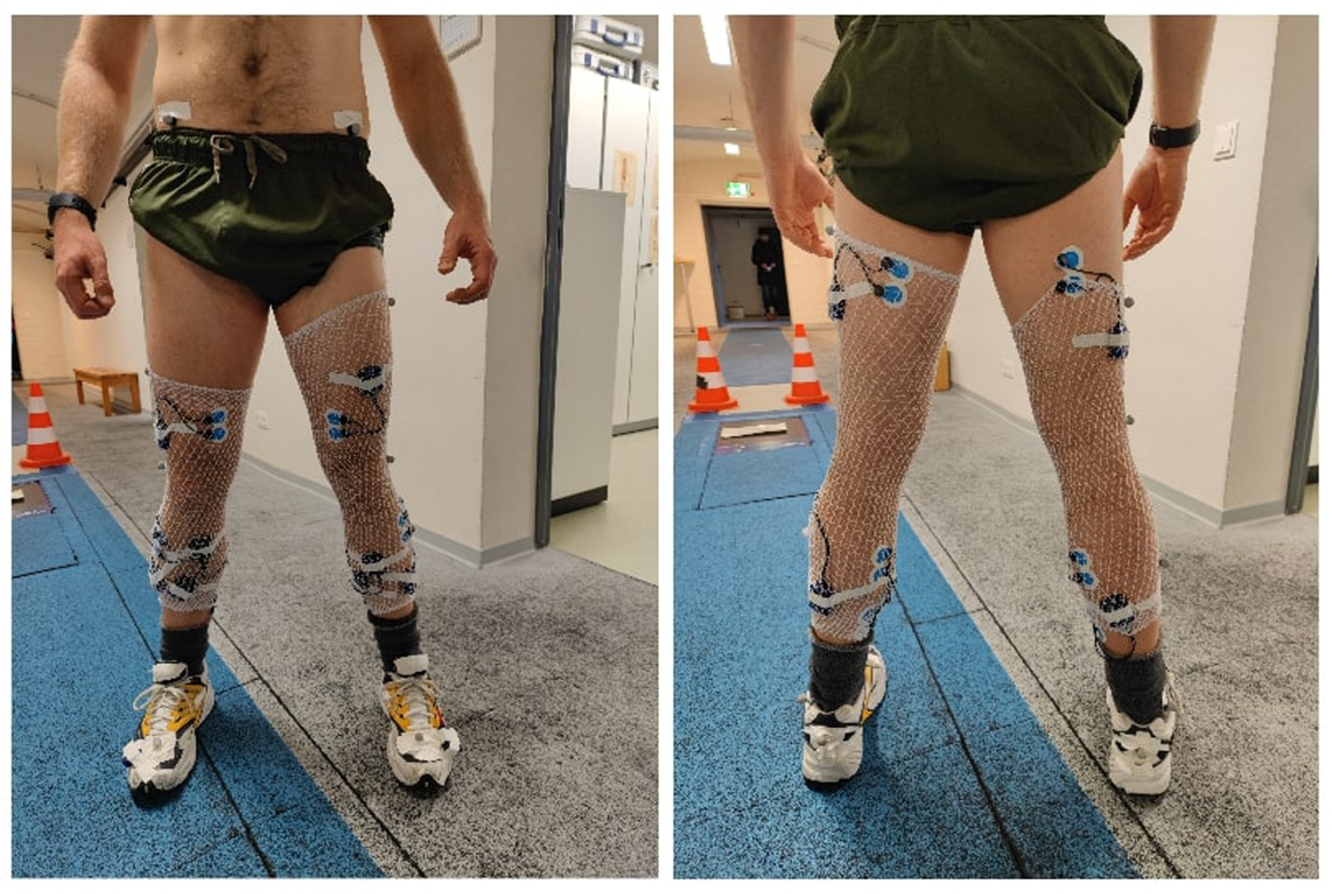
Illustrated, the anterior (left picture) and posterior view (right picture) of the lower-limb EMG setup. Electrodes were applied on both legs, and according to the SENIAM guidelines (43). In total, fourteen muscles were assessed, including M. tibialis anterior, M. peroneus longus, M. soleus, M. gastrocnemius medialis, M. vastus medialis, M. biceps femoris, and M. gluteus maximus. Note that the M. gluteus maximus Is Not pictured, for reasons of discretion. To affix the EMG boxes, tubular stockings were worn by participants. The two accelerometers can also be seen, one affixed to the heel of each shoe.

**Figure 2:**
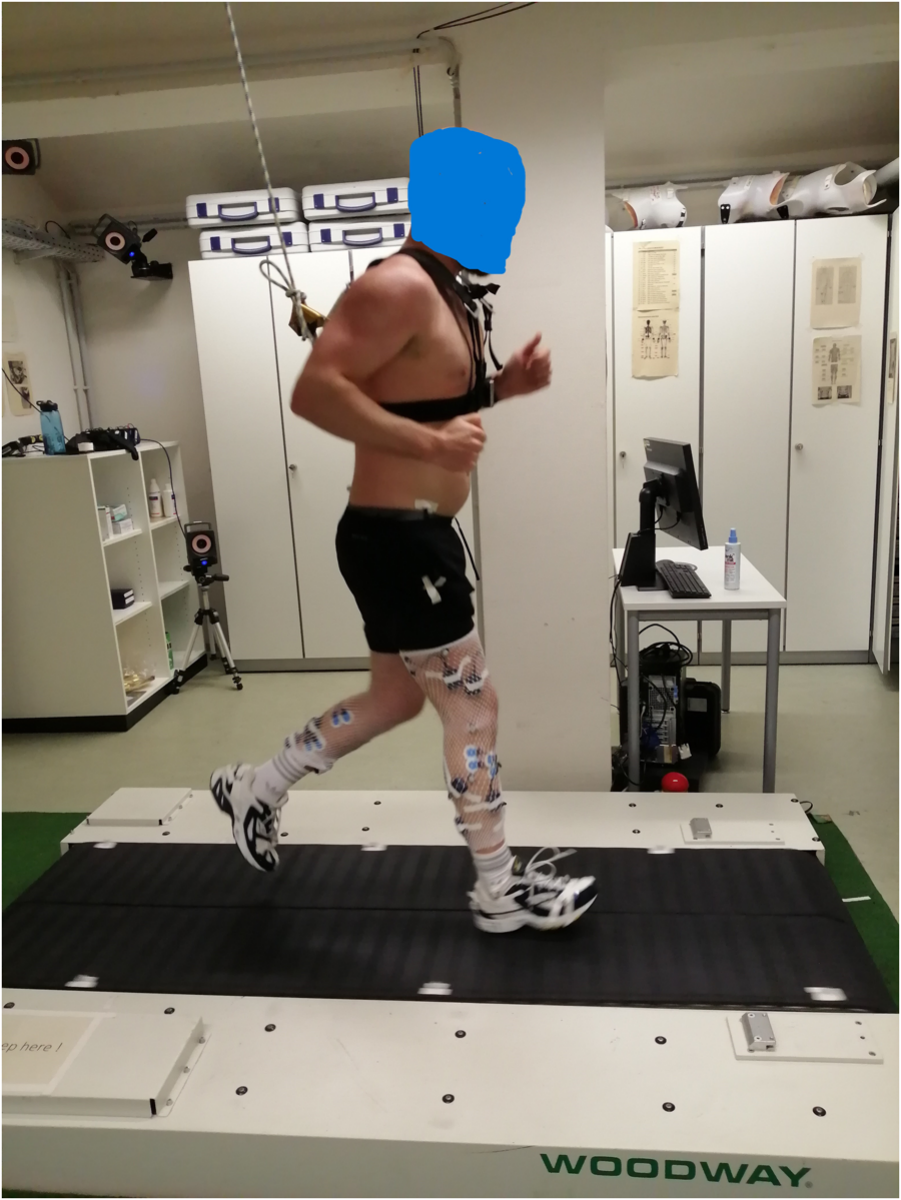
A participant seen running on the split-belt treadmill used for the perturbation protocol. The participant Is attached to an emergency brake system, *via* a chest harness.

The precise aspects of this technical setup have previously been detailed elsewhere (26). A custom split-belt treadmill [Woodway, Germany; for technical details see (26):] was used to initiate decelerative perturbation impulses of 2 m/s (40 m/s^2^) for each of the treadmill belts (right and left). Baseline velocity was 2.5 m/s (9 km/h). These parameters were selected based upon initial pilot testing, whereby perturbations that were maximally/optimally rapid, powerful, and well-timed were targeted, whilst still considering the safety of the participants and technical capacities of the treadmill. Additionally, previous work on the same treadmill displayed that decelerative perturbation impulses appear to elicit greater amplitudes in EMG activity (23, 40). Perturbations were superimposed [amplitude:2 m/s, duration:100 ms (50 ms deceleration; 50 ms acceleration)] and controlled *via* a custom software (stimuli, pfitec, biomedical systems, Germany). A load cell embedded beneath the right-hand belt of the treadmill (megatron, Max 5kN, Range ± 10 mV, Soema DAD141.1 weight indicator) was used to detect heel strike/initial foot contact events (threshold load: 10 kg), whereby any impact greater than 10 kg triggered the programmed perturbations to run. Perturbations were programmed to occur 150 ms after heel strike (delay), meaning participants were perturbed during approximately the mid-stance of gait (41). Perturbations were triggered by right heel strike events, for direct triggering of the right side and indirect triggering of the left by adding an estimate of step length based upon data extracted from the familiarisation trial, to target an execution of 150 ms post right and left foot initial contact. A 3D motion capture system (Vicon MX T10S, 13 cameras, 500 Hz, Vicon, Oxford, UK) was utilised to measure the movement of the treadmill belts (10 markers), and a further 2 markers were placed on the heel of each shoe at a standardised height (42). In addition, an acceleration sensor (Myon320s, myon AG, Switzerland) was placed on both shoes, to measure foot position and velocity. As a safety precaution, all participants wore a chest harness connected to an emergency stop. Furthermore, surface electromyography (sEMG) was applied in both legs, to enable measurement of muscle activity. Muscles measured included: M. tibialis anterior (TA), M. peroneus longus (PL), M. soleus (Sol), M. gastrocnemius medialis (GM), M. vastus medialis (VM), M. biceps femoris (BF), and M. gluteus maximus (GM). Bipolar EMG electrodes [2 cm inter-electrode distance, pre-gelled (Ag/AgCl), typeP-00-S, Ambu, Mediocotest, Denmark] were positioned with reference to the SENIAM guidelines (43). A wireless EMG capture system (band-pass filter: 5–500 Hz, gain:5.0, overall gain:2500, sampling frequency:4,000 Hz; Myon320, RFTD-32, myon AG, Switzerland) was used for recording. The EMG setup is visualised in Figure 1, and the treadmill setup is visualised in Figure 2.

### 2.3. Protocol

Each participant received a pair of standardised shoes. Firstly, participants ran for 3 min at a velocity of 2.5 m/s (9 km/h) in a familiarisation trial. After a short break, participants then completed the 8-minute running protocol at the same velocity of 2.5 m/s (9 km/h). During the protocol, 30 superimposed perturbations were implemented (15 each side). The perturbations were randomised by side and occurred at time intervals of a minimum of 10 s between each individual perturbation, to limit the effects of prior stumbling on the proceeding perturbations (14). In addition, participants were asked to estimate their subjective levels of pain before and after the perturbation protocol (Numerical Rating Scale 0–10, NRS) (44), and their Rating of Perceived Exertion (6–20, RPE) (45). For purposes of reliability testing, after a two-week washout period participants repeated the protocol for a second time identically. Moreover, data on physical activity levels of the participants was collected *via* the International Physical Activity Questionnaire—Short Form (IPAQ) (46). During the running trials, a subjective visual assessment was performed of running strike patterns, to categorise participants as either “rearfoot”, or “forefoot”. This visual assessment was later corroborated in the 3D motion analysis data.

### 2.4. Data analysis

An integration of kinematic data from the treadmill belts and shoes, combined with accelerometer data, was used to identify heel strike/foot initial contact events in both feet of each participant. Essentially, kinematic data from the vertical position of the heel marker was matched to visible impact spikes in the heel accelerometer data, which enabled identification of heel strike events (47). With this information, perturbation events could be located within the kinematic data (Vicon software, Nexus Version 2.6), and characteristics of the treadmill perturbations were extracted based upon change in velocity of the treadmill belts: amplitude (m/s), perturbation delay (ms) and perturbation duration (ms). EMG data were processed on the same software used for data capture (4th order moving average filter, IMAGO process master, pfitec, biomedical systems, Germany). The data was full wave rectified and manually checked for signal artefacts (<5% of all data). Data deemed unsuitable was discarded. Triggers were generated manually at the initiation of the perturbation impulse, located *via* the accelerometer and 3D motion capture data. These triggers defined the start of the perturbation, and all following statistical analysis were based upon this single event trigger. For definition, the standing leg directly perturbed by and in contact with the treadmill belt was assigned as the “leading leg”, whereas the contralateral swinging leg was defined as the “trailing leg”. Data from the IPAQ was analysed and rated according to established guidelines (46), producing an output of physical activity in metabolic equivalent minutes per week (MET min/Week).

### 2.5. Statistical analysis

To assess the validity of the perturbations, an average of the perturbation characteristics was calculated (mean ± SD) and the recorded group average characteristics were compared to the pre-programmed settings with a simple percentage error calculation (PE%) [(recorded value – preset value) / preset value*100]. Additionally, intra-individual variability of the recorded perturbations was calculated with coefficient of variation (CV)% (SD/mean*100) (48). Inter-session reliability for the perturbations was calculated utilising test-retest variability (TRV%: (|xi − yi |/ 0.5 (xi + yi) * 100), where xi represents the amplitude/delay/duration values of the 1st measurement and yi are those of measurement 2 for the subject i). Bland-Altman analysis with limits of agreement (LoA, bias ± 1.96*SD) was also determined (48). These outcomes related to the perturbation characteristics are additionally listed in Table 1, to enable easier comprehension. EMG perturbation amplitudes for the leading leg (mean ± SD) were calculated (4th order moving average filter, IMAGO process master, pfitec, biomedical systems, Germany) (root mean square, RMS) by normalising the average activity of 200 ms time windows post-perturbation initiation, to 200 ms time windows commencing 150 ms after heel strike during unperturbed running gait (21, 26, 49). EMG amplitudes for the trailing leg (mean ± SD) were calculated using the same method as in the leading leg, except average activity of the swinging contralateral leg was used. Latencies of muscle onset (mean ± SD) were evaluated for the leading leg only, by way of an automatic detection method [onset criteria: rise of the EMG signal above 2 SD of baseline level (50, 51)]. This data was also checked manually *via* visual inspection, to ensure plausibility and mitigate detection error. An intraclass correlation coefficient (ICC 3,1 =  two-way mixed, single measure) was calculated (52), comparing the onset latencies for each muscle and side, between the two measurement timepoints to test for the reliability of the results. Data for the RPE and NRS pain scores was reported descriptively (mean ± SD). The IPAQ was also evaluated descriptively (mean ± SD), and participants were then categorized into “high”, “moderate”, or “low” levels of physical activity.

**Table 1:** A comprehensive list of the outcomes reported relating to the programmed and recorded perturbations. Outcomes below the thick dotted line are statistical calculations, that were performed on the extracted raw perturbation characteristics outcomes (which are the three variables above the thick dotted line).

PERTURBATION OUTCOMES (UNIT)	DEFINTION/FORMULA
AMPLITUDE (m/s)	Refers to the maximum oscillation in the decelerative impulse velocity of the treadmill belts, relative to a baseline velocity of 9 km/h (2.5 m/s). Preset amplitude = 2 m/s (40 m/s^2^)
DELAY (ms)	Defined as the amount of period of time programmed between initial foot contact with the belt, and initiation of perturbation impulse. Delay preset = 150 ms
DURATION (ms) PERCENTAGE ERROR CALCULATION (%)	Refers to the length of the perturbation impulse (50 ms deceleration to approximately 0.5 m/s belt velocity; 50 ms acceleration returning to baseline velocity). Duration preset total: 100 ms = (recorded value – preset value) / preset value*100)
COEFFICIENT OF VARIATION (%)	= (SD/mean*100). Calculated as intra-individual variability, for each participant, within-session and between individual recorded perturbations.
TEST-RETEST VARIABILITY (%)	= (|*x*i−*y*i |/ 0.5 (*x*i + *y*i) * 100), where *x*i represents the amplitude/delay/duration values of the 1st measurement and yi are those of measurement 2 for the subject i). Calculated for inter-session reliability, between measurement 1 and 2.
BLAND-ALTMAN ANALYSIS (m/s or ms)	[Bias and Limits of agreement (LoA; bias ± 1.96*SD)]. Calculated between session for each of the perturbation variables, showing the systematic bias between measurements and corresponding limits of agreement.

## 3. Results

### 3.1. Participant physical activity levels and running strike patterns

Data from the IPAQ showed that participants engaged in an average 4524 ± 2736 MET min/week of physical activity. Of these participants, 75% were classified in the “high” category of physical activity, with the remaining 25% classified as “moderate”. Regarding running strike patterns, it was observed that all participants exhibited a rearfoot running strike pattern.

### 3.2. Validity and reliability of perturbation characteristics

Of the programmed perturbations, on average 10.1 ± 1.9 perturbations were detectable on the right side and 11.1 ± 1.8 perturbations were detectable on the left. Due to a technical issue, the 3D motion analysis data for one participant at one M2 timepoint had to be discarded. The results for the validity of the perturbation protocol can be seen in Table 2. Mean differences between the targeted and observed perturbations on the left side were 0.1 m/s for amplitude, 45 ms for delay, and 22 ms for duration of stimulus. Mean differences between the targeted and recorded perturbations on the right side reached 0.1 m/s for amplitude, 32 ms for delay, and 22 ms for duration of stimulus.

Results regarding the reliability of the perturbation setup can be seen in Table 3. TRV for the left-sided perturbation characteristics ranged from 6.4%–7.4%, Whereas TRV for the right-sided perturbation characteristics ranged between 12.3%–16.6%.

**Table 2:** Validity of the perturbation characteristics for directly triggered (right side) and indirectly triggered (left side) perturbations, with recorded values matched to intended presets.

PARAMETERS	UNITS	PRESET	RECORDED LEFT SIDE (MEAN ± SD)	PE% LEFT SIDE	CV% LEFT SIDE	RECORDED RIGHT SIDE (MEAN ± SD)	PE% RIGHT SIDE	CV% RIGHT SIDE
AMPLITUDE	[m/s]	2.0	1.9 ± 0.1	5%	76.8%	1.9 ± 0.2	5%	74.7%
DELAY	[ms]	150	105 ± 2	30%	29.2%	118 ± 2	21.3%	19.5%
DURATION	[ms]	100	78 ± 1	22%	27.9%	78 ± 1	22%	26.7%

Coefficient of variation % (CV%), Percentage error (PE%).

**Table 3:** Reliability of the perturbation characteristics, calculated between the two measurement time points.

PARAMETERS	UNITS	PRESET	TRV% (LEFT) (MEAN ± SD)	TRV% (RIGHT) (MEAN ± SD)	BLA (LEFT) (BIAS ± 1.96*SD)	BLA (RIGHT) (BIAS ± 1.96*SD)
AMPLITUDE	[m/s]	2.0	7.0 ± 5.0%	13.9 ± 12.9%	0.0 ± 0.3	0.1 ± 0.7
DELAY	[ms]	150	6.4 ± 6.0%	12.3 ± 10.2%	0 ± 17	4 ± 40
DURATION	[ms]	100	7.4 ± 4.8%	16.6 ± 16.9%	2 ± 13	1 ± 35

Test-retest reliability (TRV%, mean ± SD), Bland-Altman analysis with limits of agreement (BLA, mean ± SD).

### 3.3. Amplitudes and latencies of muscular reflex responses

An overview of EMG amplitudes in both the leading and trailing limbs following perturbations when normalised to unperturbed gait, can be viewed in Table 4. In the left limb, EMG amplitudes ranged from 175 ± 141% (GM)–454 ± 359% (VM) in the leading leg, and from 118 ± 22% (TA)–221 ± 182% (VM) in the trailing leg. In the right limb, EMG amplitudes were between 190 ± 112% (GM)–343 ± 356% (BF) in the leading leg, and 124 ± 54% (TA)–238 ± 169% (VM) in the trailing leg.

**Table 4:** Average EMG amplitudes following perturbation in the leading and trailing legs. Data displayed Is only extracted from a single measurement time point (M1).

	AMPLITUDES (RMS%, MEAN ± SD)			
MUSCLES	Leading Leg (Left)	Trailing Leg (Left)	Leading Leg (Right)	Trailing Leg (Right)
TA	257 ± 142%	118 ± 22%	247 ± 97%	124 ± 54%
PL	229 ± 133%	160 ± 78%	286 ± 200%	172 ± 93%
SOL	211 ± 128%	142 ± 59%	223 ± 129%	184 ± 91%
GM	175 ± 141%	150 ± 109%	190 ± 112%	127 ± 104%
VM	454 ± 359%	221 ± 182%	252 ± 191%	238 ± 169%
BF	253 ± 156%	167 ± 57%	343 ± 356%	161 ± 65%
GMAX	310 ± 240%	190 ± 112%	208 ± 144%	209 ± 127%

TA, M. tibialis anterior; PL, M. peroneus longus; Sol, M. Soleus; GM, M. gastrocnemius medialis; VM, M. vastus medialis; BF, M. biceps femoris; GMax, M. gluteus maximus. Root mean square % (RMS%).

**Figure 3:**
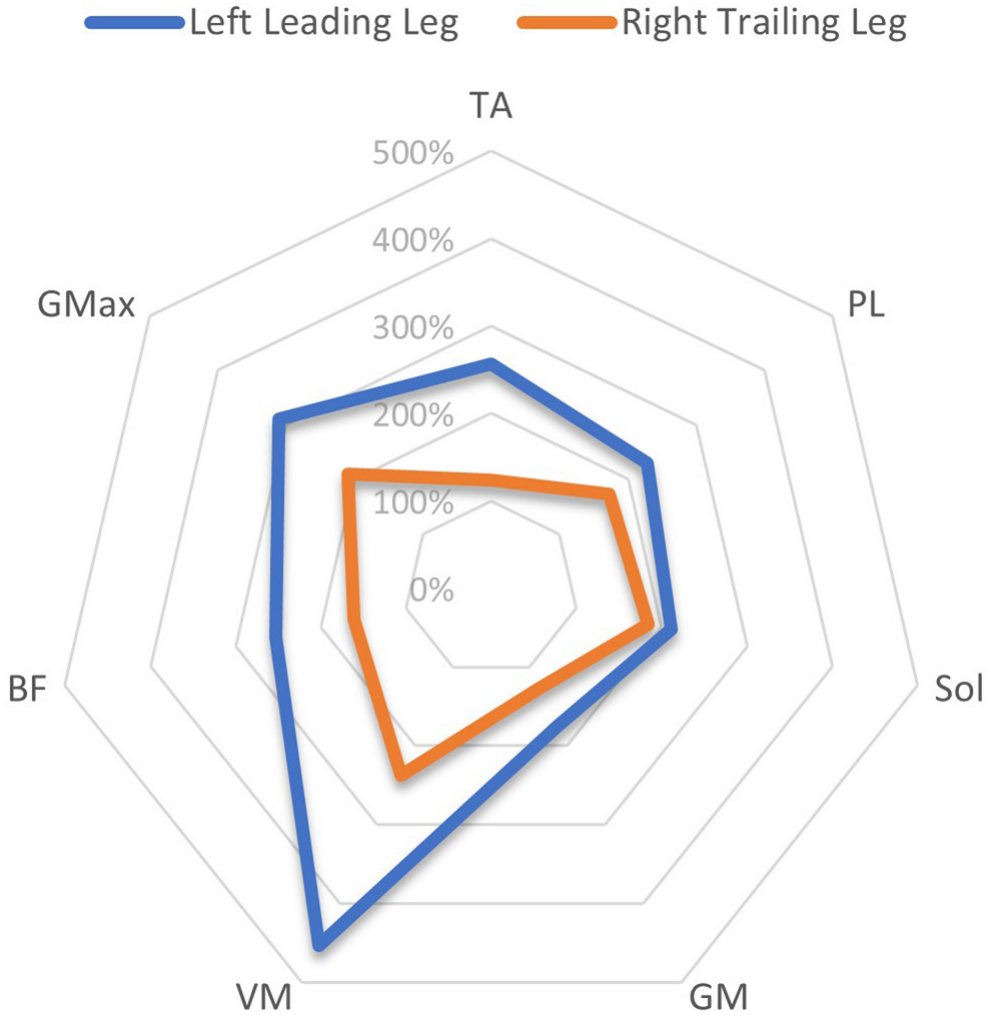
A radar chart of EMG RMS% mean perturbation amplitudes of the left leading and right trailing leg following left-sided perturbations, When normalised as a ratio to the same phase as unperturbed gait. This ratio Is expressed as a percentage of activity, considering the amplitude levels 200 ms after the perturbation onset/equivalent phase during unperturbed gait. Note that points plotted towards the edge of the graph represent a greater magnitude of relative muscle activity, in response to the perturbations. The centre of the chart represents 0%. Distal musculature = TA, M. tibialis anterior; PL, M. peroneus longus; Sol, M. Soleus; GM, M. gastrocnemius medialis; Proximal musculature = VM, M. vastus medialis; BF, M. biceps femoris; GMax, M. gluteus maximus. Data displayed Is only extracted from a single measurement time point (M1).

**Figure 4:**
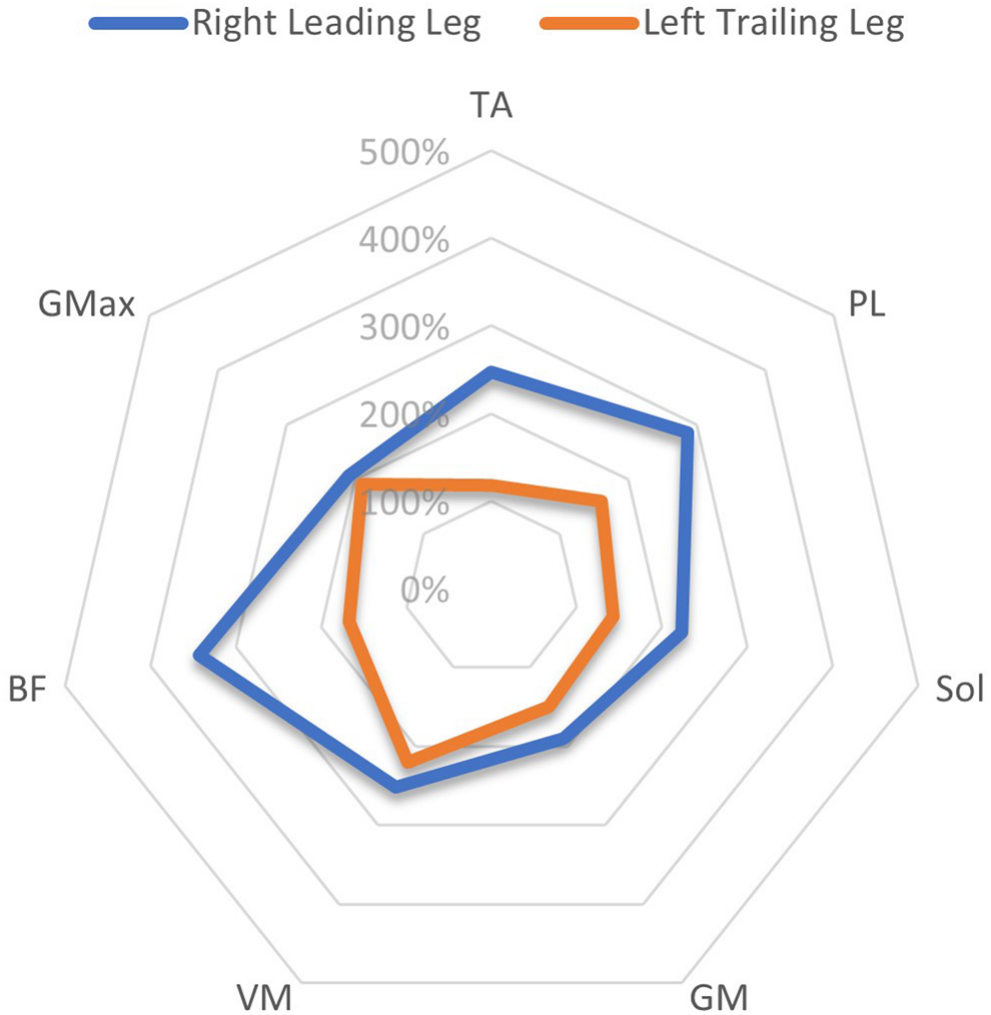
A radar chart of EMG RMS% mean perturbation amplitudes of the right leading and left trailing leg following right-sided perturbations, When normalised as a ratio to the same phase as unperturbed gait. This ratio Is expressed as a percentage of activity, considering the amplitude levels 200 ms after the perturbation onset/equivalent phase during unperturbed gait. Note that points plotted towards the edge of the graph represent a greater magnitude of relative muscle activity, in response to the perturbations. The centre of the chart represents 0%. Distal musculature = TA, M. tibialis anterior; PL, M. peroneus longus; Sol, M. Soleus; GM, M. gastrocnemius medialis; Proximal musculature = VM, M. vastus medialis; BF, M. biceps femoris; GMax, M. gluteus maximus. Data displayed Is only extracted from a single measurement time point (M1).

A comparison of the EMG amplitude activity between the leading and trailing legs for both the left and right-sided perturbations is illustrated in Figure 3 (left-sided perturbations) and Figure 4 (right-sided perturbations).

**Figure 5:**
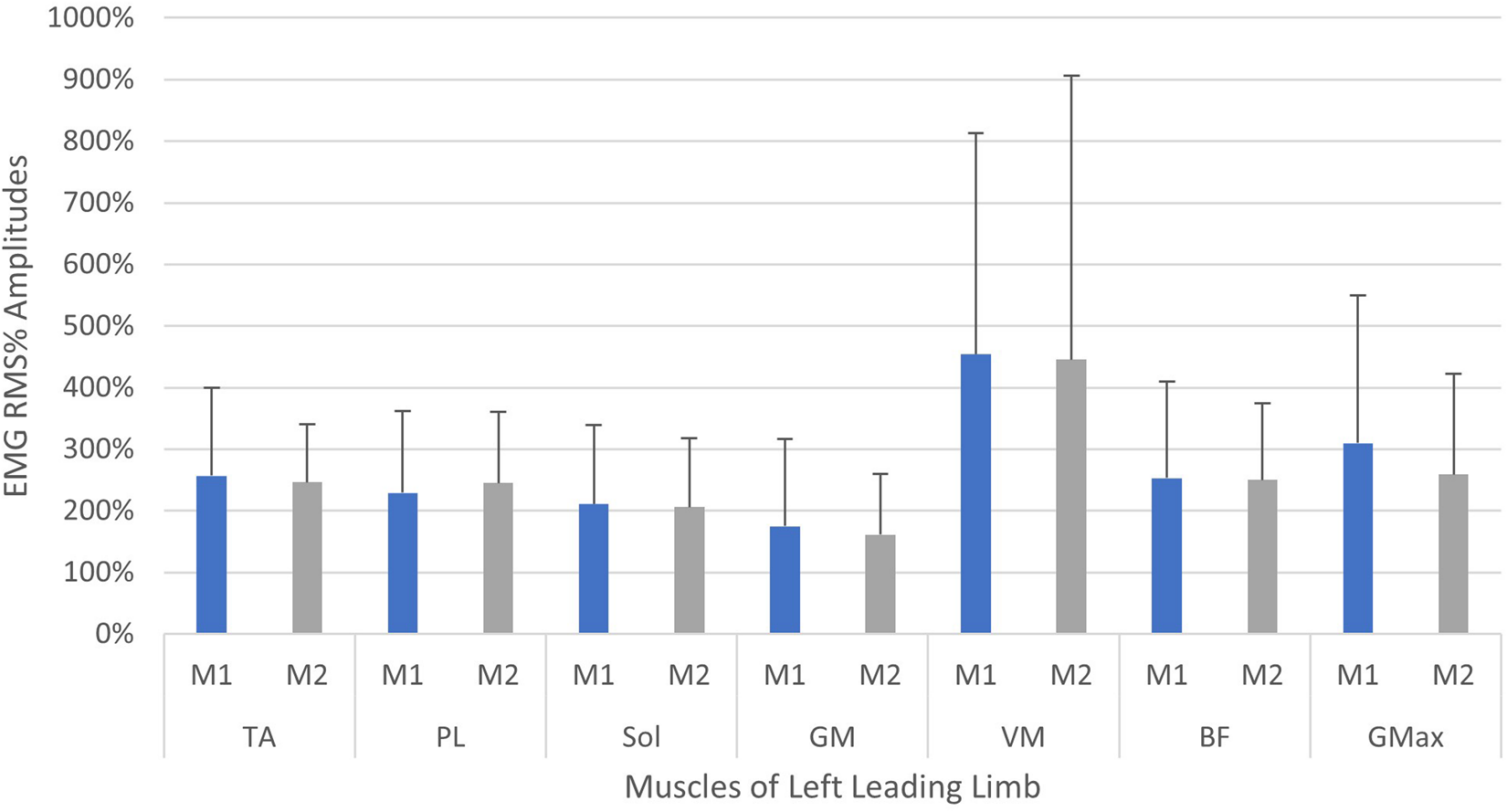
Neuromuscular reflex activity of leading leg, 200 ms following left-sided perturbations, shown between two measurement time points (M1/M2, test-retest approximately 2 weeks apart). Muscles of left limb—TA, M. tibialis anterior; PL, M. peroneus longus; Sol, M. soleus; GM, M. gastrocnemius medialis; VM, M. vastus medialis; BF, M. biceps femoris; GMax, M. gluteus maximus. Root mean square % (RMS%).

**Figure 6:**
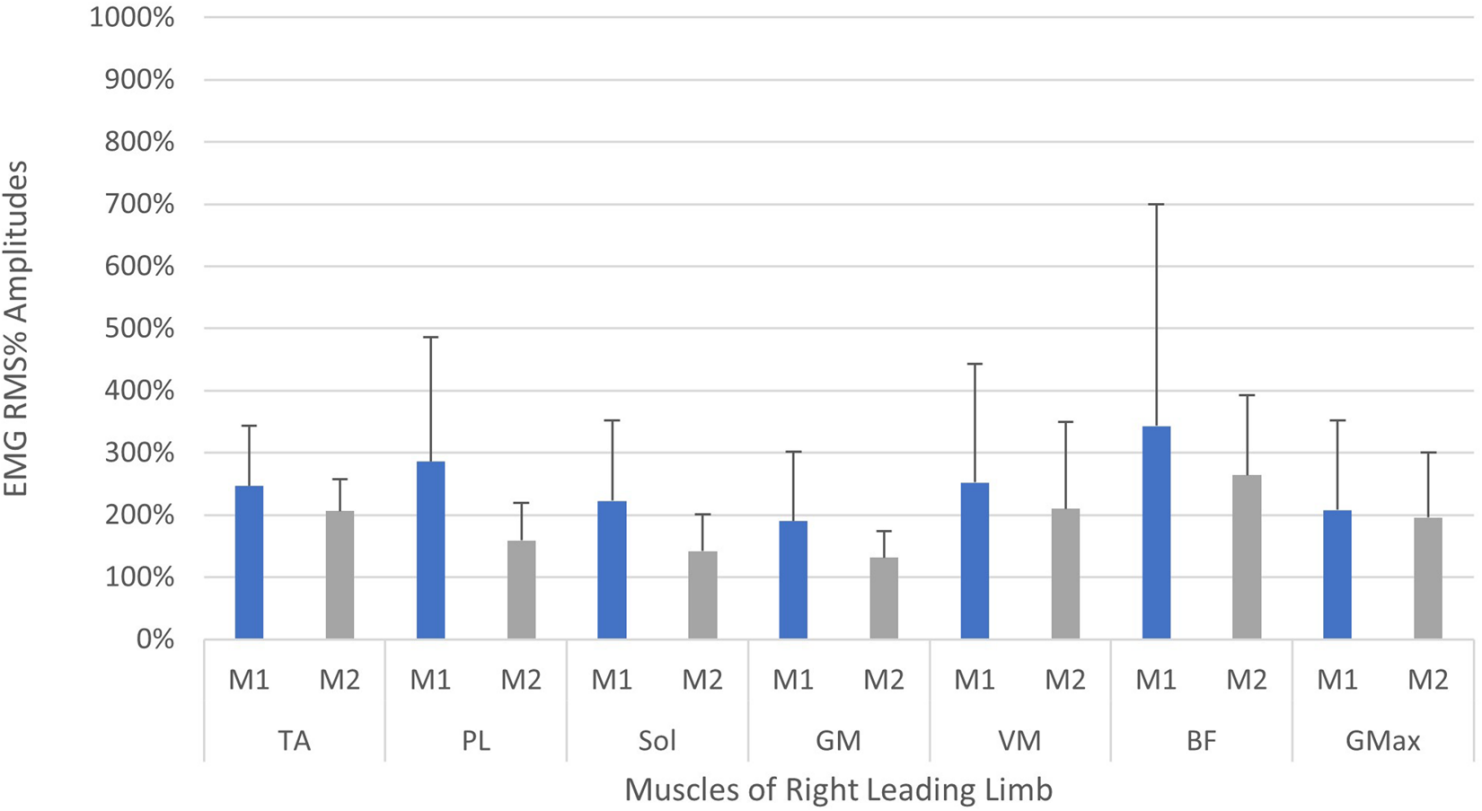
Neuromuscular reflex activity of leading leg 200 ms following right-sided perturbations, shown between two measurement time points (M1/M2, test-retest approximately 2 weeks apart). Muscles of right limb—TA, M. tibialis anterior; PL, M. peroneus longus; Sol, M. Soleus; GM, M. gastrocnemius medialis; VM, M. vastus medialis; BF, M. biceps femoris; GMax, M. gluteus maximus. Root mean square % (RMS%).

A comparison of EMG amplitudes (mean ± SD) between the two measurement time points is displayed in Figures 5, 6. Muscular activity is shown for both limbs in response to the perturbations, and for the leading legs only.

Onset of muscle latencies could only be calculated for the TA and BF muscles (see Table 5), as the data for other muscles was not sufficient due to difficulty in identifying the onset signal. Latencies ranged from 109 ± 12–116 ± 23 ms in the TA of both legs, and from 128 ± 49–157 ± 20 ms in the BF of both legs.

**Table 5:** Onset latencies of the leading legs (mean ± SD, ms) post-perturbation (right leg = right-sided perturbation, left leg = left-sided perturbation) measured at two time points (M1 and M2).

MUSCLE	RIGHT LEG M1	RIGHT LEG M2	ICC	LEFT LEG M1	LEFT LEG M2	ICC
TA	115 ± 10	116 ± 23	0.32	109 ± 12	110 ± 20	0.40
BF	157 ± 20	129 ± 43	0.91	128 ± 49	129 ± 44	0.78

ICC, Intraclass Correlation Coefficient. TA, M. tibialis anterior; BF, M. biceps femoris.

### 3.4. Subjective RPE and pain

On average, participants rated the perturbation trial as 14 ± 2.3 on an RPE scale, meaning they felt the trial was between “somewhat hard” and “hard”. On an NRS pain scale between 0 and 10, participants rated their subjective pain after the trial as being 0.1 ± 0.3 on average, changing minimally from 0.3 ± 0.8 pre-trial.

## 4. Discussion

This study aimed to investigate the technical validity and reliability of a perturbed treadmill running protocol, and to assess corresponding neuromuscular reflex activity. The sample of participants collected were an active (“moderate” to “high” on the IPAQ) and relatively young population, who all exhibited a rearfoot running strike pattern. When comparing preset and recorded mean results for perturbation characteristics, accuracy of the protocol was variable, showing a percentage error rate between 5% (amplitude) and 30% (duration) and indicating moderate and variable validity of the perturbations. Results of test-retest revealed good reliability on the left side (TRV < 7.5%), whilst the perturbation protocol was slightly less reliable on the right (TRV < 17%). As evidenced in the EMG data, the protocol clearly elicited muscular reflex activity in the lower limbs, with more activity apparent in the leading than the trailing leg. Therefore, the hypothesis that the protocol would be reliable between sessions could only be accepted for the left-sided perturbations, as the reliability of perturbations on the right-side fell slightly below acceptable standards. The hypotheses that the left and right-sided perturbations would exhibit similar validity, and that the protocol would provoke reflex activity, could be accepted based upon data within the current study.

### 4.1. Validity and reliability of perturbation characteristics

Whilst some studies have examined the effects of other types of perturbations during running, for example, accelerative impulses modifying running velocity (53), or mediolateral perturbations at the trunk (54), it appears that no other study has successfully produced distal belt perturbations both rapid and powerful enough (20), to provoke reflex responses of the lower extremities (22). Therefore, there is no literature currently available to compare the relative validity and reliability of the protocol in the current study, so comparisons with data from walking protocols were made. A recent study was able to execute perturbations *via* a split-belt treadmill during walking, with an onset timing error of 5.2% (55). This is less than the mean percentage error reported in the current study (delay error = 21%–30%) and implies an inability of the treadmill to produce the desired timing characteristics. This might reflect a technical limitation of the treadmill, or represent the difficulty of accurately delivering perturbations during a running task, which differs significantly to walking (56, 57). Timing of the perturbations is critical to ensure that the stimulus is delivered in the intended gait phase (14), and in the current protocol a disturbance during the mid-stance of running gait was targeted (41). On average, perturbations were timed 105 ms (left) and 118 ms (right) post-heel contact, which is earlier than expected but would still be within an acceptable physiological range (41, 56). Based upon this, it can be stipulated with reasonable confidence that the perturbations were delivered within a suitable timeframe, whereby weight acceptance of the landing limb has begun prior to onset of perturbation. Other walking protocols have executed perturbations *via* the treadmill belts using lower amplitudes of 1.2 m/s (22), and much higher amplitudes up to 5 m/s (10), with a longer stimuli duration of 400 ms (22) and 540 ms (10) respectively. When studying reflex responses, short-timed duration stimuli are thought to be crucial, so as to minimise the ongoing effects of mechanical perturbation on recorded muscle activity (20–22). The average duration of perturbation stimuli in the current study was 78 ms on both sides, which is 22% lower than the intended value. However, given that reflex muscle activity has been shown to occur less than 70 ms post-perturbation (22), this shorter duration might still be appropriate, and arguably better if stimulation of reflex responses is the desired aim. Intra-individual variability (CV%) of the recorded perturbations ranged from 19.5%–76.8%, showing large variation in the nature of the applied perturbations, especially in the domain of amplitude (76.8% - left, 74.7% - right). The values obtained are similar to those reported in a previous investigation (26), although variation was slightly higher on the left side (e.g., CV delay = 29.2% - left; 19.5% - right), perhaps due to the indirect triggering of the perturbation *via* a step length estimation method. This highlights the need to be sensitive to the apparent variability within the nature of the executed perturbations and may necessitate yet to be determined threshold criteria, relating to the classification of individual perturbations. The authors can only speculate as to the reasons for this variability within the protocol, but it could be attributed to a technical failure in the communication between the treadmill load cell, computer and utilised software, and the treadmill belts. Alternatively, the natural variation in foot placement and running dynamics may interface differently with the technical setup throughout the protocol, resulting in the apparent variability of the applied stimuli that were recorded.

The only study assessing the reliability of any such protocol was conducted by Engel et al., (2017), which reported test-retest values of TRV < 6%, and bias <3 ms (delay, duration) and ∼0 m/s (amplitude) for walking perturbations. Results in the current study revealed good reliability on the left side, indicated by TRV < 7.5% and bias <2 ms (delay and duration) and ∼0 m/s (amplitude). Results for the right side were somewhat less reliable, whereby TRV was below 17%, and bias was <4 ms (delay and duration) and =0.1 m/s (amplitude). Reliability is inferior in the current running study compared to walking (21), probably due to the inherent difficulties of applying the perturbations at a running velocity, whereby baseline treadmill velocity is 250% higher and there is an increased potential for foot misplacement. Future studies should aim to report on the validity and reliability of conducted perturbed running protocols so that collective solutions can be shared and understood, especially considering the current deficit of information in this area of research (8). Exactly how valid and reliable such protocols need to be remains an open question, and hopefully the data conveyed in the current study can lay the groundwork for this discussion. Ultimately, in an ideal case researchers could develop a protocol capable of delivering rapid, powerful, well-timed, and controlled perturbations during running in a reproducible way; a task which was partly achieved within this research project. We hope that future investigations will bring us closer to this goal.

### 4.2. Muscular reflex responses

The current study showed that the programmed perturbations can provoke reflex muscle activity in the lower extremities, as displayed in the EMG data (Tables 4, 5). Muscle activation was most apparent in the leading leg, with the muscles TA, BF, VM, and Gmax at the highest levels, whilst the GM was least active (Table 4). This corroborates findings from previous trials with similar protocols in running (26) and walking (21). EMG amplitudes were higher in response to the perturbations in the leading leg, when compared to the trailing leg (see Figures 3, 4). This supports previous studies which have investigated muscle activity and changes in kinematics during perturbed running (19, 28, 36). The trailing leg is known to play a role in the maintenance of balance during perturbed walking (51, 58), and the results of the current study suggest that it is also active during running perturbations; whilst to a lesser extent than the leading leg. Data indicate that the trailing leg muscles of VM, BF, and Gmax were particularly active, potentially alluding to proximal muscle firing to alter the position of the entire lower extremity segment, and thereby effectively compensating the perturbation impulses. Recent research utilising the same protocol as the current study but with a different cohort, indicated that the trailing leg was chiefly responsible for kinematic compensations in response to the perturbations (27). This could appear to contradict the findings of the current study, which found greater muscle activity in the leading limb. However, this might be explained by the greater forces demanded of the leading leg when met with the direct impulse of the perturbation from the treadmill belt, with co-contraction of multiple muscles enabling effective stabilisation of the lower-limb segment (23). This behaviour seems opposed to the trailing limb, which appears to adjust its kinematic position in space, though with minimal muscle activation whilst in an open kinetic chain. Nevertheless, much more research is needed to explore this phenomenon. Especially considering that the primary aim of this study was to investigate the reliability and validity of the protocol setup, and these secondary findings are strictly exploratory in nature. To confirm or reject this phenomenon would require an adequately statistically powered future study with more specific aims relating to the muscle activity outcomes.

The specific design of the perturbation paradigm is likely to have produced distinct neuromuscular responses within the participants. Namely, the perturbations were programmed to decelerate for 50 ms and then accelerate for 50 ms, returning the treadmill to baseline velocity. This difference in the direction of the treadmill and hence foot displacement, is likely to have brought about diverse muscle activity patterns (20, 40). It could be speculated that the responses measured within the current study are primarily indicative of a reaction to the decelerative component of the perturbations, considering this was the initial stimulus, and when referring to the higher EMG activity of the BF and GMax musculature (see Table 4). These muscles could be profiled as eccentric stabilisers in this motor pattern, supported by co-contraction of the VM to stabilise the pelvis and knee. However, responses to the accelerative phase of the perturbations cannot be excluded, although one would expect to see such responses later within the 200 ms window of activity that was analysed. It is difficult to isolate the effects of the declarative and accelerative perturbation components within the current paradigm design, due to the necessity of the accelerative component to return to baseline treadmill velocity. Essentially, extraction of the precise nature of the EMG responses to whichever phase of the perturbation was truly delivered is a process that requires much further research. Future studies might consider dissecting the EMG data into distinct timepoints to partition relevant reactions to specific directions of the perturbations (40), perhaps supported by kinematic data to verify individual event phases. The 200 ms window for EMG data analysis selected in the current study was based upon previous evidence suggesting reflex activity up to 180 ms after onset of an unexpected perturbation (23, 49, 60), though it could be argued that activity in the later end of this time-window is more voluntary in nature and no longer true reflex activity (60). According to data in the current study, we measured onset latencies as late as 170 ms after perturbation in the BF (means for this data seen in Table 5).

**Figure 7:**
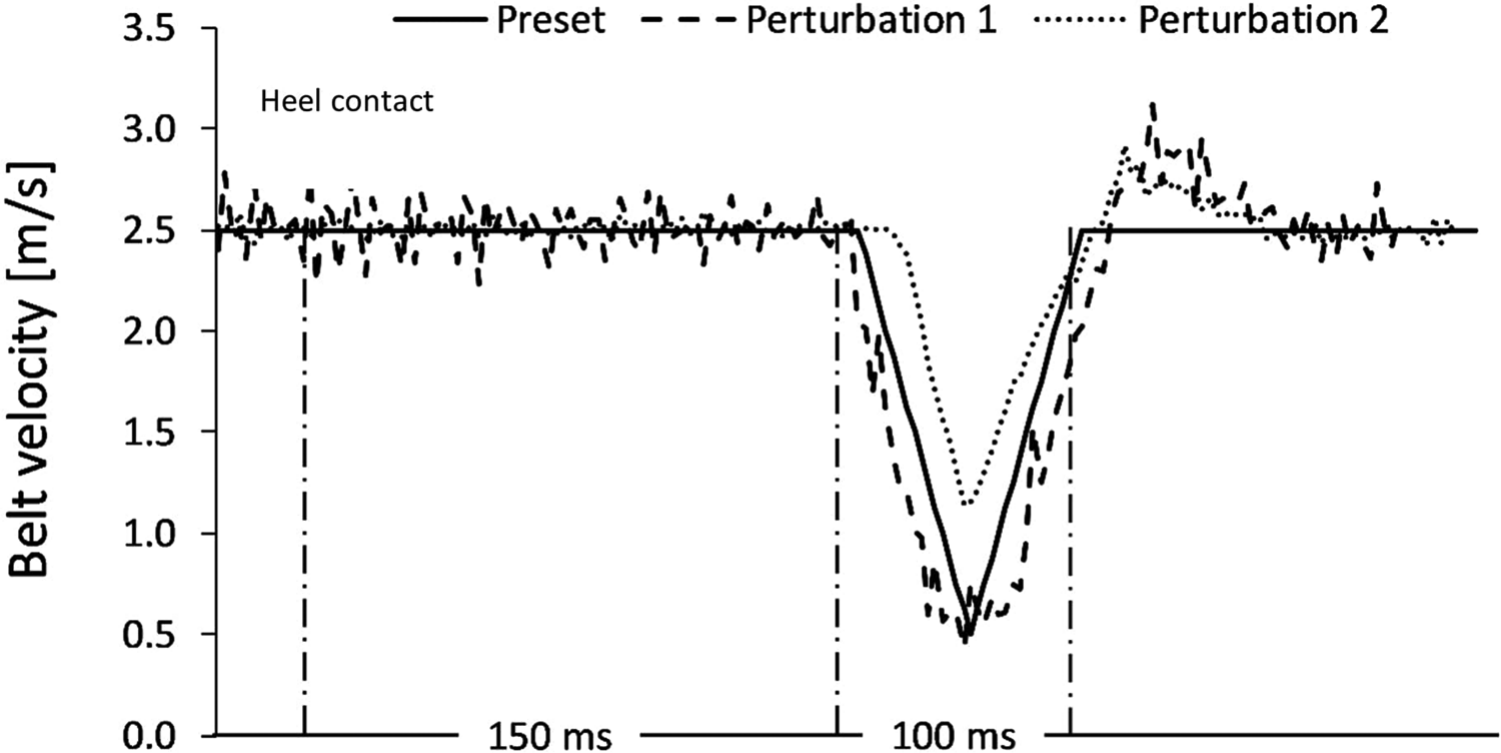
A representation of two extracted perturbations, plotted against the preset (intended) characteristics of the programmed perturbations. The black solid line Is representative of the intended characteristics. Amplitude (m/s) of the treadmill belt Is displayed vertically on the *y*-axis. The 150 ms window post-heel strike indicates the delay of the perturbation. The following 100 ms represents the assumed duration. Perturbation 1 achieved the intended characteristics, whereas perturbation 2 initiated later and less powerfully than intended, with a shorter duration.

When considering the size of the EMG amplitudes and variability of the data (SD), a general trend indicated a reduction in the overall activity and variation of motor output in the leading leg, between the first and second measurements (see Figures 5, 6). The trend is particularly apparent in the right leg (see Figure 5) and this might illustrate a motor learning strategy, whereby participants habituated their responses to the perturbations *via* a training effect (19, 61). A separate study has shown that a longer training intervention of running with perturbations can induce adaptations in EMG profiles (19), reporting reduced variability in muscle activity after 18 training sessions. However, the nature of the perturbations applied in this study were substantially different, utilising elastic tubing strapped to the legs of participants (19), and the effects of motor learning on EMG signalling remain highly controversial (61, 62). The variability in the EMG data captured during the first measurement, may be indicative of a novel stimulus inducing “exploratory” activity of the lower limb musculature (63). This “exploratory” sensory-motor nervous system activity is seemingly dynamic in nature, which supports learning strategies based upon error prediction and proprioceptive feedback i.e., reinforcement learning. It has been posited that large motor variability in the initial stages of adaptation to a novel task is crucial and inherent to the learning process (63). Indeed, one study investigating arm pattern trajectories reported that not only is variability somehow inherent to learning a task, but actually predicts motor learning ability, at least within this sample of healthy participants (64). It might therefore be pertinent to treat motor variability not as “noise” in the signal, but indeed part of the signal itself (63, 65). These learning effects should be factored into any test-retest study designs when measuring EMG reflex responses longitudinally and would particularly have implications for intervention studies aiming to measure the causal effects of a specific intervention. Furthermore, it is interesting to observe that the EMG variability was less attenuated in the left limb (see Figure 5) when compared to the right leg (see Figure 6). This could reflect the increased intra-individual variability in the timing of the perturbations in the left leg in relation to the right leg (see Table 2, CV% delay), which implies less predictability in the nature of the applied perturbations on the left side. It could be speculated that this wider range of stimuli violated expectation-based learning models, and participants were therefore unable to learn a sufficient neuromuscular response within the timeframe of the initial session (63, 64). In other words, participants were still in the “exploratory” phase of motor learning in the second session, and unable to “exploit” prior experiences, meaning adaptations and potentially attenuated EMG reflex responses were not as present (64). It must however be noted that the intra-individual variability of perturbations in both legs was generally quite high across characteristics of the perturbations (see Table 2 and Figure 7). It could be stipulated that this variance indicates a bidirectional human-machine interaction, which the data within the current study cannot fully comprehend. Postural adjustments and learning strategies throughout the protocol, might result in different behaviour of the delivered perturbations due to altered timing/foot placement in relation to the embedded force plate trigger. Such an interaction would need to be measured and potentially excluded, to truly identify physiological motor adaptations within participants.

Onset muscle latencies for the leading leg TA and BF ranged between 109 and 157 ms in the current study, whereby muscle onset appeared to be earlier in the TA muscle than in the BF. Onset latencies for the TA have been reported in walking trials (21, 66), with a range of 65–88 ms respectively. This onset is at least 21 ms earlier than TA latency in the current trial (109–116 ms), perhaps explained by the specific perturbation setup or a distinct outcome during a running task. Latencies for the BF muscle were longer, and this is supported by previous evidence during walking (38), whereby a delay of up to 175 ms was reported in the medial hamstring muscle, which is up to 18 ms longer than values reported in the current trial. Whether the delayed hamstring onset is a task-specific response to perturbations of this type or a more general finding, should be the topic of future scientific experiment (67). Analysis of onset latencies between the two measurements indicated good-excellent reliability for the BF muscle (ICC = 0.78–0.91), and poor reliability for the TA muscle (ICC = 0.32–0.40) (52), meaning the protocol could be applied confidently in future studies for the BF muscle but values for the TA should be interpreted cautiously. Future work should attempt to identify the reason for this discrepancy, which could be attributed to the nature of the assessment protocol or the innate physiological variability of TA reflex activity in response to the task.

### 4.3. Limitations

Finally, some limitations of the study should be acknowledged. Data in this study were collected on a convenience sample size of twelve participants, tested twice two weeks apart. This small sample size should be factored in when interpreting the outcomes of this research, especially regarding findings in the EMG data. However, given the time-intensive nature of conducting such research, and the potent lack of investigation in this area, we believe that the findings still provide valuable insight for those interested in human gait and motor control. Furthermore, the participants in this sample were a relatively young and active population, therefore caution should be warranted when applying these results to different populations of interest, for example elderly sedentary people. All twelve participants were heel-strikers, and this will have obvious implications on the kinetics and kinematics of our included sample (68). Data from the current study can therefore only be applied to a heel-strike pattern running population, and further studies investigating other strike patterns such as forefoot should be conducted. Additionally, running velocity was set to a single treadmill speed, and not individualised. This could have resulted in an “unnatural” running style for some participants, although this method was chosen to allow for standardised perturbations executed at a specific phase of gait (10, 14). Additionally, the current method does not allow for perturbations in the swing phase of gait, where “trip-like” disturbances often occur (21).

### 4.4. Conclusions

Notwithstanding the aforementioned limitations, to the authors knowledge this is the first study to show that perturbations can be implemented reliably *via* the belts of a split-belt treadmill during running, despite the technical challenges. The validity of the protocol is decisively mixed, especially regarding the amplitude and duration of the perturbations. The delivery of well-timed and powerful stimuli during running with such a methodology is technically demanding and will require more work from future researchers to improve the accuracy of the protocol. Despite the technical limitations of the protocol, the authors still believe that this setup could be applied usefully and yield interesting scientific findings, given the requisite knowledge of these fundamental issues. The protocol successfully provoked neuromuscular reflex responses of the lower extremities and can assess onset of BF muscle reflex activity in response to the perturbations reliably. The remaining exploration of EMG data when comparing amplitudes between the leading and trailing legs, and between sessions provides interesting avenues for future research, however these data were strictly preliminary and should be handled as such. Knowledge gained from application of such protocols could be applied in a wide range of fields, for example in developing understanding of balance during gait and especially fall risk (1, 2) which is a significant global health burden and the leading cause of fatal and nonfatal injuries in adults over the age of 65 years (69, 70). Attempts to study balance recovery strategies during human gait could result in targeted intervention programs which aim to prevent falls in at risk populations (8, 69). Additionally, the protocol presented in the current study offers the ability to study neuromuscular reflex responses in a functional task such as human gait, which could be considered to have more ecological validity than protocols that rely on less functional tasks to elicit reflex responses (8, 71). Reflexes are considered to represent a critical aspect of human wellbeing and performance, indicative of both musculoskeletal and neurological health (7, 8, 40). Reflexes are also known to diminish with age (7, 69), and are also associated with other conditions, such as non-specific chronic low back pain (23), and neurological conditions such as stroke (25). The protocol described in the current research or those similar to it, could be utilised to enhance our understanding of reflex responses in such populations and improve management of patients in orthopaedic and physical therapy settings. Previous research has indicated that reflexes are trainable in elderly people (22), though the clinical relevance of such interventions in other populations is still unclear. Furthermore, the details of postural control during human gait are still reasonably uncharacterised, and comprehension of this phenomenon will be vital for any future research in robotics, and the potential for augmented limbs and prosthetics in humans (1–3, 34). The current protocol could be used to map out the mechanisms by which humans recover from external ground perturbations and maintain equilibrium (3). Future investigations might apply the protocol in healthy and clinical populations, to enhance our understanding of postural control in humans during running. This could be with reference to acute adjustments to the perturbation protocol within session, or regarding chronic adaptations in response to training or therapeutic interventions, which could be tested longitudinally.

## Data Availability

The raw data supporting the conclusions of this article will be made available by the authors, without undue reservation.
